# An examination of perceived partner phubbing in romantic relationships across various variables

**DOI:** 10.3389/fpsyg.2026.1846877

**Published:** 2026-06-23

**Authors:** Müzeyyen Soyer, Seçil Ömür Sünbül

**Affiliations:** Department of Educational Sciences, Mersin University, Mersin, Türkiye

**Keywords:** partner phubbing, marital status, relationship duration, gender, age, smartphone use, technoference

## Abstract

**Background:**

Partner phubbing, defined as ignoring one’s romantic partner by attending to a mobile phone during face-to-face interactions, has become an increasingly important issue in romantic relationships. This study examined perceived partner phubbing across gender, marital status, age, and relationship duration.

**Methods:**

A correlational survey design was employed with 446 adults currently involved in a romantic relationship. Data were collected using the Partner Phubbing Scale. Independent samples t-tests, one-way analyses of variance (ANOVAs), and multiple linear regression analyses were conducted to examine differences and predictors of perceived partner phubbing.

**Results:**

Women reported significantly higher levels of perceived partner phubbing than men, and married individuals scored higher than single individuals. Analyses with respect to age indicated that young adults reported lower levels of perceived partner phubbing than older age groups, with perceptions increasing with age. Perceived partner phubbing also differed according to relationship duration, with individuals in relationships lasting 5–10 years reporting the highest levels. Multiple regression analyses revealed that gender and being in a relationship for 5–10 years were significant predictors of perceived partner phubbing.

**Discussion:**

The findings suggest that demographic and relational characteristics play an important role in shaping perceptions of partner phubbing. These results contribute to the growing literature on technology-related behaviors in romantic relationships and have important implications for couple therapy and digital wellbeing interventions.

## 1 Introduction

With the widespread integration of smartphones into everyday life, the nature of face-to-face interactions has undergone a substantial transformation. This transformation directly affects fundamental relational processes in romantic relationships, such as emotional intimacy, relationship satisfaction, and commitment (Wang et al., 2025). Associated with the intensive use of digital technologies, partner phubbing is defined as a multidimensional interpersonal interaction problem in which an individual, despite being physically present with their romantic partner, directs their attention—either consciously or automatically—to their smartphone, and this behavior is perceived by the partner as neglect, devaluation, or being pushed into the background (Roberts and David, 2016). Partner phubbing is regarded as a behavioral pattern that disrupts mutual interest, responsiveness, and emotional availability in romantic relationships (Miller-Ott and Kelly, 2015; Beukeboom and Pollmann, 2021; Nie et al., 2025). This behavior includes actions such as checking the phone while the partner is speaking, texting, spending time on social media, or prioritizing notifications, all of which diminish the quality of face-to-face interaction (Chotpitayasunondh and Douglas, 2018).

The literature indicates that individuals who are exposed to phubbing by their partners experience increased perceptions of rejection, which negatively affects relationship quality and emotional intimacy (Przybylski and Weinstein, 2013; Tiryaki Göksu et al., 2025; Yam, 2023; Karaman and Arslan, 2024; Rahmawati and Yasmin, 2025; Sharafat et al., 2025). In this process, individuals may interpret their partner’s attention to the phone as a sign of reduced interest and commitment toward them (Carnelley et al., 2025). This perceptual evaluation may in turn weaken trust, intimacy, and mutual commitment, which are among the core components of romantic relationships (Chotpitayasunondh and Douglas, 2018; Halpern and Katz, 2017; Doğru et al., 2025). Indeed, Dikdere and Türkarslan (2024) demonstrated that perceived partner phubbing is negatively associated with relationship satisfaction, and that this relationship becomes even stronger through mediating variables such as loneliness.

Perceived partner phubbing affects not only relationship dynamics but also individuals’ psychological well-being. Repeated exposure to phubbing may trigger feelings of loneliness, exclusion, and diminished self-worth, while also reinforcing perceptions of social exclusion (David and Roberts, 2017). In addition, this experience has been reported to be associated with depressive symptoms (McDaniel and Coyne, 2016; Yaldızcı, 2025; Liang et al., 2025). Furthermore, experiences of neglect by one’s partner may increase individuals’ attachment to social media, and orientation toward digital environments may function as a compensatory coping strategy (David and Roberts, 2017). In this context, partner phubbing is considered an important indicator of the negative psychosocial effects of modern technology on romantic relationships (Roberts and David, 2016). Therefore, this phenomenon should be understood not merely as a habit of technology use, but as a complex interpersonal process that plays a decisive role in emotional availability, perceived value, and relational trust within romantic relationships.

Although the existing literature has demonstrated the negative relational outcomes of partner phubbing, the phenomenon has rarely been grounded in a comprehensive theoretical framework (Roberts and David, 2016; Chotpitayasunondh and Douglas, 2018; McDaniel and Coyne, 2016). To better understand partner phubbing as a complex interpersonal process, it can be interpreted through multiple complementary theoretical perspectives, including interpersonal communication theory, social exchange theory, and digital communication frameworks (Przybylski and Weinstein, 2013; Turkle, 2011; Krasnova et al., 2016).

From the perspective of interpersonal communication theory, romantic relationships are sustained through mutual responsiveness, active listening, and emotional availability (Reis and Shaver, 1988; Laurenceau et al., 1998). Perceived partner responsiveness, in particular, has been identified as a central mechanism underlying intimacy and relationship satisfaction (Reis et al., 2004; Maisel et al., 2008). In this context, partner phubbing can be conceptualized as a disruption of core communication processes, as it signals inattentiveness and reduces perceived partner responsiveness (Przybylski and Weinstein, 2013; Miller-Ott and Kelly, 2015). When one partner directs attention toward a smartphone instead of engaging in face-to-face interaction, this behavior may be interpreted as a form of relational disengagement, thereby weakening emotional connection and intimacy (Halpern and Katz, 2017; Beukeboom and Pollmann, 2021).

In addition, social exchange theory (Blau, 1964; Thibaut and Kelley, 1959) provides a useful framework for understanding how individuals evaluate their relationships based on perceived rewards and costs. Relationship satisfaction and commitment are shaped by the balance between these perceived rewards and costs (Rusbult, 1980; Rusbult et al., 1998). Within this framework, partner phubbing may function as a relational cost, as it diminishes the perceived benefits of interaction (e.g., attention, validation, emotional support) while increasing feelings of neglect and dissatisfaction (Roberts and David, 2016; David and Roberts, 2017). Over time, repeated exposure to such costs may lead individuals to reassess the overall value of the relationship, potentially undermining commitment and relational stability (Rusbult, 1980; Dainton, 2001).

Furthermore, within the context of technology-mediated communication and digital interaction frameworks, phubbing can be understood as a form of “technoference,” referring to the intrusion of technology into interpersonal interactions (McDaniel and Coyne, 2016). Digital communication theories suggest that the presence of mobile devices alters interaction norms, attention allocation, and social expectations (Turkle, 2011; Baym, 2015). Empirical studies have shown that even the mere presence of a mobile phone can reduce the quality of face-to-face conversations and perceived closeness between interaction partners (Przybylski and Weinstein, 2013). In romantic relationships, this shift may create a paradox in which physical presence coexists with psychological absence (Turkle, 2011). As a result, partner phubbing may not only reflect individual technology use habits but also broader socio-cultural transformations in communication patterns (Castells, 2010; Griffiths, 2018).

Taken together, these theoretical perspectives suggest that partner phubbing should not be considered merely as a behavioral habit, but rather as a multidimensional interpersonal phenomenon embedded in communication processes, relational evaluations, and digital interaction norms (Chotpitayasunondh and Douglas, 2018; Beukeboom and Pollmann, 2021; McDaniel and Coyne, 2016; Przybylski and Weinstein, 2013). This theoretical framework provides an important conceptual basis for understanding how individuals interpret partner phubbing experiences and under which conditions these perceptions may vary (Reis et al., 2004; Rusbult et al., 1998). Considering that interpersonal responsiveness, relational cost–benefit evaluations, and digital interaction norms may differ across individuals, it is reasonable to expect that perceptions of partner phubbing may also vary depending on individual and relational characteristics (Turkle, 2011; Baym, 2015; Dainton, 2001).

Building on this theoretical foundation, the present study aims to examine whether perceived partner phubbing differs according to variables such as gender, marital status, age, and relationship duration, and further investigates the extent to which these demographic and relational variables predict perceived partner phubbing. Although the existing literature has addressed perceived partner phubbing in relation to various variables, the question of the individual and relational characteristics under which this behavior varies has been relatively neglected (Roberts and David, 2016; David and Roberts, 2017; Chotpitayasunondh and Douglas, 2018). In particular, the role of variables such as gender, age, marital status, and relationship duration in perceived partner phubbing has been examined in a limited and fragmented manner (Çizmeci, 2017; Halpern and Katz, 2017; Beukeboom and Pollmann, 2021). In other words, the literature has primarily focused on the outcomes of phubbing, whereas comparatively less attention has been paid to the question of among whom and under what conditions phubbing is more pronounced (McDaniel and Coyne, 2016). In addition, findings regarding potential variations across different cultural and demographic samples remain insufficiently systematic (Khodabakhsh and Ong, 2021; Koşmaz, 2024). By examining whether perceived partner phubbing differs according to these demographic variables, this study seeks to fill this gap in the literature and to provide comprehensive evidence regarding the groups in which perceived partner phubbing is more pronounced.

## 2 Method

### 2.1 Research design

In this study, the current state of perceived partner phubbing in romantic relationships was described, and whether it differed according to various variables was examined. The study was conducted using a descriptive and correlational survey design, one of the quantitative research methods. The survey model is a research approach that aims to describe a past or existing situation as it is (Karasar, 2020).

### 2.2 Study group

The study group consisted of 446 individuals who were involved in a romantic relationship. The sample was determined through convenience sampling, a non-probability sampling method. In terms of gender distribution, 80.94% of the participants were women and 19.06% were men. Data were collected via an online survey platform distributed through social media channels. Participation was voluntary and no compensation was provided. Regarding marital status, 56.28% of the participants were married and 43.72% were single. When age categories were examined, 23.77% of the participants were between 19 and 25 years old, 41.70% were between 26 and 35 years old, and 34.53% were 36 years old and above. With regard to relationship duration, 22.87% of the participants reported being in a relationship for 1–12 months, 21.08% for 1–3 years, 9.42% for 3–5 years, 18.83% for 5–10 years, and 27.80% for 10 years or more.

*A priori* power analysis conducted using G*Power 3.1 indicated that a minimum sample size of 107 participants was required to detect a medium effect size (*f* = 0.25) in a one-way ANOVA with five groups, at a power of 0.80 and *α* = 0.05. The present sample of 446 participants substantially exceeded this threshold, providing adequate statistical power for all planned analyses.

### 2.3 Data collection instruments

In this study, the Partner Phubbing Scale was used alongside a Personal Information Form developed by the researcher in order to determine participants’ demographic characteristics, such as gender, marital status, age, and relationship duration.

#### 2.3.1 Partner Phubbing Scale

The Partner Phubbing Scale, developed by Roberts and David (2016), was designed to measure the level of phubbing perceived from a partner among individuals who are married or involved in a romantic relationship. The scale is a single-factor, 9-item, 5-point Likert-type instrument intended to assess perceived phubbing behaviors. The minimum possible score on the scale is 9, whereas the maximum possible score is 45. Higher scores indicate higher levels of perceived partner phubbing. In the original study, the reliability analysis yielded a Cronbach’s alpha coefficient of 0.92. The instrument was adapted into Turkish by Çizmeci (2017), and subsequent analyses reported a Cronbach’s alpha coefficient of 0.89.

In the present study, the psychometric properties of the Partner Phubbing Scale were re-examined, and the reliability analysis demonstrated that the scale had a high level of internal consistency (Cronbach’s alpha = 0.859). The corrected item-total correlations ranged from 0.30 to 0.719, and the factor analysis suitability tests indicated that the data were highly appropriate for factor analysis (KMO = 0.922, Bartlett’s *χ*^2^(36) = 1,566, *p* < 0.001). The results of the parallel analysis supported a single-factor structure, and this single factor explained 44.22% of the total variance. The single-factor structure was further tested through confirmatory factor analysis (CFA), and the results indicated an excellent model-data fit [*χ*^2^(27) = 52.52, *p* = 0.002; CFI = 0.984; TLI = 0.978; RMSEA = 0.046 (90% CI = 0.027–0.064); SRMR = 0.030; GFI = 0.973; AGFI = 0.955]. These findings demonstrate that the Turkish version of the Partner Phubbing Scale is a valid and reliable measurement instrument and that it is capable of measuring partner phone use in romantic relationships within a unidimensional structure.

### 2.4 Data analysis

The research data were analyzed using SPSS 25.0. Prior to the main analyses, the dataset was examined in terms of normality assumptions, outliers, and missing data. In order to describe the participants’ demographic characteristics, including gender, age, and relationship duration, frequency and percentage distributions were calculated. In addition, descriptive statistics for the Partner Phubbing Scale scores, including mean, standard deviation, minimum, maximum, skewness, and kurtosis values, were reported. To assess whether the data were normally distributed, the Shapiro–Wilk normality test was conducted. In addition, skewness and kurtosis coefficients within the range of ±1.5 were considered indicative of an acceptable normal distribution (Tabachnick and Fidell, 2013). In order to evaluate the psychometric properties of the Partner Phubbing Scale, reliability analysis, exploratory factor analysis (EFA), and confirmatory factor analysis (CFA) were conducted, respectively. To test the reliability of the scale, the Cronbach’s alpha internal consistency coefficient was calculated, and item-total correlations were examined. In interpreting the reliability coefficient, the criterion of *α* ≥ 0.70 was adopted (Nunnally and Bernstein, 1994). Before conducting the factor analysis, the suitability of the data for factor analysis was evaluated using the Kaiser–Meyer–Olkin (KMO) measure of sampling adequacy and Bartlett’s test of sphericity. A KMO value above 0.60 and a statistically significant Bartlett’s test result (*p* < 0.05) were taken as evidence that the data were appropriate for factor analysis (Tabachnick and Fidell, 2013). To determine the factor structure of the scale, exploratory factor analysis was performed using the principal components analysis method. In determining the number of factors, the parallel analysis method was used, and factors with eigenvalues greater than 1 were taken into consideration (Horn, 1965). In the interpretation of factor loadings, values of 0.30 and above were considered acceptable (Büyüköztürk, 2018). To verify the factor structure identified through EFA, confirmatory factor analysis was subsequently performed. In evaluating model-data fit, the following criteria were used for the fit indices: for CFI and TLI, values of ≥0.90 were considered indicative of acceptable fit and ≥0.95 of excellent fit; for RMSEA, values of ≤0.08 indicated acceptable fit and ≤0.06 indicated excellent fit; for SRMR, values of ≤0.08 indicated acceptable fit and ≤0.05 indicated excellent fit; and for GFI and AGFI, values of ≥0.90 were considered acceptable and ≥0.95 were considered excellent. To test whether partner phubbing scores differed across demographic variables such as gender, marital status, age, and relationship duration, an independent samples t-test and one-way analysis of variance (ANOVA) were employed. When the ANOVA results indicated significant differences, the Tukey HSD *post hoc* test was used to determine between which groups these differences occurred. Finally, a multiple linear regression analysis was conducted to identify the predictors of perceived partner phubbing. Gender, marital status, age group, and relationship duration were entered into the model as predictor variables, and the perceived partner phubbing total score was entered as the dependent variable. Categorical variables were dummy coded prior to entry; female gender, single marital status, the 19–25 age group, and the 1–12 months relationship duration group were designated as reference categories.

## 3 Results

In this section, the findings obtained from the analysis of the data collected in line with the purpose of the study are presented. First, descriptive statistics regarding the scores obtained from the Partner Phubbing Scale were examined in order to determine the participants’ levels of perceived partner phubbing. Subsequently, within the scope of the sub-problems of the study, the relationships and differences among the variables were analyzed.

### 3.1 Findings on participants’ levels of perceived partner phubbing

In order to determine the overall level of perceived partner phubbing among the individuals who participated in the study, descriptive statistics related to the total scores obtained from the Partner Phubbing Scale were calculated and are presented in Table 1.

**Table 1:** Descriptive statistics for total scores on the Partner Phubbing Scale.

Variable	*N*	Mean	Standard deviation	Minimum	Maximum	Skewness	Kurtosis
Phubbing	446	24.61	7.25	9	43	0.32	−0.38

As shown in Table 1, descriptive statistics for the total scores on the Partner Phubbing Scale were calculated for the 446 participants. The mean perceived partner phubbing score was 24.61 (SD = 7.25). Although the possible score range of the scale is 9–45, the observed scores in the present study ranged from 9 to 43, indicating a moderate level of perceived partner phubbing among the participants.

In addition, the median score was 24 and the mode was 23. The closeness of the median and mode to the mean suggests that the distribution was relatively symmetrical. This interpretation is further supported by the skewness coefficient (0.32) and the kurtosis coefficient (−0.38), both of which fell within the acceptable range of ±1.5, indicating that the data were normally distributed.

### 3.2 Gender differences in perceived partner phubbing

An independent samples t-test was conducted to determine whether perceived partner phubbing differed by gender. The results of the analysis are presented in Table 2.

**Table 2:** Independent samples *t*-test results for perceived partner phubbing scores by gender.

Group	*N*	M	SD	*t*	df	*p*	Effect size
Women	361	25.44	7.05	5.13	444	<0.001	0.62
Men	85	21.08	7.06				

M, mean; SD, standard deviation; df, degrees of freedom.

Prior to the analysis, the assumptions of homogeneity of variances and normality were examined. Levene’s test indicated that the variances were equal across groups, and the Shapiro–Wilk tests showed that the assumption of normality was met for both subgroups. As shown in Table 2, women reported significantly higher perceived partner phubbing scores than men.

### 3.3 Marital status differences in perceived partner phubbing

An independent samples *t*-test was conducted to determine whether perceived partner phubbing differed by marital status. The results of the analysis are presented in Table 3.

**Table 3:** Independent samples *t*-test results for perceived partner phubbing scores by marital status.

Group	*N*	M	SD	*t*	df	*p*	Effect size
Married	251	25.69	7.54	−3.60	444	<0.001	0.34
Single	195	23.23	6.62				

M, mean; SD, standard deviation; df, degrees of freedom.

Prior to the analysis, the assumptions of homogeneity of variances and normality were examined. Levene’s test indicated that the variances were homogeneous across groups, and the Shapiro–Wilk tests showed that the assumption of normality was met for both subgroups. As shown in Table 3, married individuals reported significantly higher perceived partner phubbing scores than single individuals.

### 3.4 Age differences in perceived partner phubbing

A one-way analysis of variance (ANOVA) was conducted to determine whether perceived partner phubbing differed by age. The results of the analysis are presented in Table 4.

**Table 4:** Descriptive statistics and one-way ANOVA results for perceived partner phubbing by age group.

Age group	*N*	M	SD	*F*	*p*	*η^2^*
19–25	106	22.47	6.80	6.21	0.002	0.027
26–35	186	25.25	7.14			
36 and older	154	25.32	7.44			

M, mean; SD, standard deviation; *F, F*-statistic; *η*^2^, eta-squared (effect size). *Post hoc* comparisons were conducted using Tukey’s HSD test.

Prior to the analysis, the assumptions for parametric testing were examined. Levene’s test indicated that the assumption of homogeneity of variances was met, and the Shapiro–Wilk tests showed that the assumption of normality was met for all age subgroups. The one-way ANOVA showed that perceived partner phubbing scores differed significantly across age groups, *F*(2, 443) = 6.21, *p* = 0.002, *η*^2^ = 0.027. Tukey’s HSD *post hoc* comparisons showed that participants aged 26–35 reported significantly higher perceived partner phubbing scores than participants aged 19–25. Similarly, participants aged 36 and older reported significantly higher perceived partner phubbing scores than participants aged 19–25. However, no significant difference was found between the 26–35 and 36 and older age groups. These findings indicate that participants aged 19–25 perceived lower levels of partner phubbing than participants in the older age groups.

### 3.5 Relationship duration differences in perceived partner phubbing

A one-way analysis of variance (ANOVA) was conducted to determine whether perceived partner phubbing differed according to relationship duration. The results of the analysis are presented in Table 5.

**Table 5:** Descriptive statistics and one-way ANOVA results for perceived partner phubbing scores by relationship duration.

Relationship duration	*N*	M	SD	*F*	*p*	*η^2^*
1–12 months	114	22.46	6.73	4.75	0.001	0.04
1–3 years	83	24.52	6.34			
3–5 years	43	24.93	6.28			
5–10 years	83	26.86	8.19			
10 years or more	123	25.04	7.48			

M = Mean; SD = standard deviation; *F* = *F*-statistic; *η*^2^ = eta-squared (effect size). Reference category for relationship duration is 1–12 months. *Post hoc* comparisons were conducted using Tukey’s HSD test.

Prior to the analysis, the assumptions for parametric testing were examined. The Shapiro–Wilk tests indicated that the assumption of normality was met across relationship duration groups. However, Levene’s test indicated a violation of the homogeneity of variances assumption; therefore, the results should be interpreted with caution. A one-way ANOVA revealed a statistically significant difference in perceived partner phubbing scores according to relationship duration, *F*(4, 441) = 4.75, *p* = 0.001, *η*^2^ = 0.04. Descriptive findings showed that participants who had been in a relationship for 5–10 years reported the highest perceived partner phubbing scores, whereas participants who had been in a relationship for 1–12 months reported the lowest scores. *Post hoc* comparisons indicated that participants in relationships lasting 5–10 years had significantly higher perceived partner phubbing scores than those in relationships lasting 1–12 months. This finding suggests that perceived partner phubbing may become more pronounced as relationship duration increases, particularly among individuals in relationships lasting 5–10 years.

### 3.6 Multiple linear regression analysis

A multiple linear regression analysis was conducted to determine whether gender, marital status, age group, and relationship duration predicted perceived partner phubbing. Before conducting the analysis, the assumptions of multiple linear regression were examined. The independence of errors assumption was evaluated using the Durbin–Watson statistic, which was 1.96, indicating no serious autocorrelation problem. The categorical predictors were dummy coded before being entered into the model. Female, single, the 19–25 age group, and the 1–12 months relationship duration group were used as the reference categories. The linearity assumption was examined through residual plots and a model specification check, and the results did not indicate a serious violation of this assumption. The normality of residuals was evaluated using the Shapiro–Wilk test and a Q–Q plot. Although the Shapiro–Wilk test was significant, visual inspection of the Q–Q plot suggested that the residuals did not show a severe departure from normality. In addition, no extreme residual outliers were detected based on standardized residuals, as no cases exceeded |*z*| > 3. Multicollinearity was examined using tolerance and variance inflation factor values. The tolerance values ranged from 0.308 to 0.950, and the VIF values ranged from 1.05 to 3.25, indicating that multicollinearity was not a serious concern. The homoscedasticity assumption was evaluated using residual plots and the Breusch–Pagan test. Influential observations were examined using Cook’s distance values. Although some cases exceeded the conventional 4/N criterion, the maximum Cook’s distance value was 0.024, which was well below 1, suggesting that no highly influential observations were present.

A multiple linear regression analysis was conducted to examine the predictive roles of gender, marital status, age group, and relationship duration on perceived partner phubbing. The 1 to 12 months category served as the reference group for relationship duration, and the 19–25 age group served as the reference for age. The overall model was statistically significant, *F*(8, 437) = 6.646, *p* < 0.001, *R^2^* = 0.108, adjusted *R^2^* = 0.092, indicating that the predictors collectively accounted for approximately 10.8% of the variance in perceived partner phubbing.

As shown in Table 6, gender was the only statistically significant predictor, *B* = −4.41, *SE* = 0.85, *β* = −0.24, *t*(437) = −5.19, *p* < 0.001, indicating that male participants reported significantly lower levels of perceived partner phubbing than female participants. Marital status (*B* = 1.52, SE = 0.97, *β* = 0.10, *t* = 1.57, *p* = 0.117), age group 26–35 (*B* = 1.21, SE = 0.95, *β* = 0.08, *t* = 1.27, *p* = 0.203), and age group 36 and above (*B* = 1.78, SE = 1.22, *β* = 0.12, *t* = 1.46, *p* = 0.145) were not significant predictors.

**Table 6:** Multiple linear regression analysis predicting perceived partner phubbing

Predictor	*B*	SE	*β*	*t*	*p*	95% CI LL	95% CI UL
Constant	22.59	0.82	—	27.45	<0.001	21.04	24.23
Gender—male	−4.41	0.85	−0.24	−5.19	<0.001	−6.07	−2.71
Marital status—married	1.52	0.97	0.10	1.57	0.117	−0.24	3.54
Age—26–35	1.21	0.95	0.08	1.27	0.203	−0.63	3.08
Age—36 and older	1.78	1.22	0.12	1.46	0.145	−0.46	4.31
Relationship duration—1–3 years	0.89	1.02	0.05	0.88	0.380	−1.15	2.87
Relationship duration—3–5 years	1.65	1.32	0.07	1.25	0.213	−1.17	3.89
Relationship duration—5–10 years	2.44	1.21	0.13	2.02	0.044	0.04	4.58
Relationship duration—10 years or more	0.32	1.39	0.02	0.23	0.819	−2.64	2.54

*N* = 446. The dependent variable was perceived partner phubbing. Female, single, the 19–25 age group, and the 1–12 months relationship duration group were used as the reference categories. CI, confidence interval; LL, lower limit; UL, upper limit; SE, standard error.

Among relationship duration predictors, only the 5 to 10 years group was a statistically significant predictor (*B* = 2.44, SE = 1.21, *β* = 0.13, *t*(437) = 2.02, *p* = 0.044), indicating that individuals in relationships of 5 to 10 years perceived significantly more partner phubbing than those in relationships of 12 months or less. The 1–3 years (*B* = 0.89, SE = 1.02, *β* = 0.05, *t* = 0.88, *p* = 0.380), 3–5 years (*B* = 1.65, SE = 1.32, *β* = 0.07, *t* = 1.25, *p* = 0.213), and 10 years or more (*B* = 0.32, SE = 1.39, *β* = 0.02, *t* = 0.23, *p* = 0.819) groups did not differ significantly from the reference group.

## 4 Discussion

This study aimed to examine perceived partner phubbing in romantic relationships in terms of various demographic variables. The findings indicated that gender, marital status, age, and relationship duration had significant effects on perceived partner phubbing.

The first finding of the study revealed that women perceived more phubbing behavior from their partners in romantic relationships. This finding is consistent with the study by Çizmeci (2017), which reported that women were exposed to phubbing more frequently than men. Similarly, Sharafat et al. (2025) found that women reported both higher levels of phubbing and lower relationship satisfaction. This difference may be explained by the fact that women tend to place greater importance on relational communication and may be more sensitive to disruptions in face-to-face interaction. In addition, men’s tendency to devote more time to technology use may increase their partners’ perceptions of phubbing. The study conducted by Wang et al. (2025) on Chinese young adults showed that partner phubbing affected relationship quality through perceived partner responsiveness, and this mediating effect was found to be significant only among women. Taken together, these findings suggest that phubbing behavior should be addressed within the framework of gender dynamics, and that such differences should be taken into account in intervention programs.

The analyses conducted in terms of marital status showed that married individuals had higher perceived partner phubbing scores than single individuals. This finding suggests that the perception of phubbing may be more common in relationships involving long-term and institutional commitment, such as marriage. This finding aligns with evidence suggesting that individuals in committed, long-term relationships tend to be more affected by their partners’ phubbing behavior (Wang et al., 2025; Koşmaz, 2024). Similarly, Koşmaz (2024) found that perceived partner phubbing differed significantly among married individuals. In the study by Wang et al. (2025) the negative relationship between partner phubbing and relationship satisfaction was found to be significant only among married adults, whereas this relationship was not significant among unmarried individuals involved in serious romantic relationships. David and Roberts (2017) also reported that married individuals were more sensitive to phubbing and that perceived partner phubbing reduced relationship satisfaction. Married couples may spend more time together and have more intertwined daily routines, which may make phone use more noticeable. In contrast, unmarried couples may be more focused on each other due to the relatively newer and more exciting nature of their relationships. Although many qualitative and quantitative studies have focused on partner phubbing, most have used a common sample consisting of romantic partners regardless of whether they were married or unmarried. However, the number of studies directly comparing single and married groups as a marital status variable remains limited. Therefore, it is believed that the findings of the present study make a valuable contribution to the literature.

Analyses regarding the age variable indicated that younger adults had lower perceived partner phubbing scores than older age groups, and that perceived partner phubbing was significantly higher among participants aged 26 and above compared to those aged 19–25; however, no significant difference was observed between the 26–35 and 36-and-older age groups, suggesting that phubbing perceptions may plateau after the mid-twenties rather than increase linearly with age. However, the existing literature presents mixed findings on this issue. For example, Beukeboom and Pollmann (2021) reported that older individuals were exposed to less partner phubbing, while Halpern and Katz (2017) and Schokkenbroek et al. (2022) similarly found a negative relationship between phubbing and age. On the other hand, Koşmaz (2024) reported that perceptions of phubbing increased among individuals aged 31 and above. Likewise, Çizmeci (2017) indicated that younger individuals perceived their partners’ phone use as less disturbing, whereas perceptions of phubbing were higher among middle-aged and older groups. In contrast, Khodabakhsh and Ong (2021) also emphasized that perceived partner phubbing decreased as age increased. The absence of a significant difference between the older age groups in the present study may suggest that perceptions of phubbing stabilize after a certain age. In addition, Wang et al. (2025) reported findings indicating that older couples perceive the effects of phubbing on relationships differently from younger age groups, whereas Şimşek and Başaran (2025) showed that the prevalence of perceived partner phubbing and its negative relational consequences partially decreased with age. Taken together, these findings suggest that the literature does not point to a fully consistent pattern, yet the present results remain broadly comparable to prior work showing that age is associated with differences in phubbing perceptions.

Analyses based on relationship duration revealed a statistically significant difference between groups and showed that perceived partner phubbing scores generally increased as relationship duration lengthened. Participants in the early months of their relationship had the lowest phubbing scores, whereas those in relationships lasting 5 to 10 years had the highest scores. A slight decrease was observed in relationships of 10 years or more. This pattern suggests that partners may show greater attentiveness to one another at the beginning of the relationship, whereas over time, routines and increased technology use may contribute to higher levels of phubbing behavior (Roberts and David, 2016; Chotpitayasunondh and Douglas, 2018). The decrease observed in very long-term relationships may be explained by couples becoming accustomed to this situation or by developing shared rules regarding technology use (McDaniel and Coyne, 2016). Although the effect size was small, indicating that the effect of relationship duration on perceived partner phubbing was limited (Cohen, 1988), the findings nonetheless emphasize the importance of regulating technology use and preserving relationship quality in long-term relationships.

In the literature, Roberts and David (2016) included relationship duration as a control variable, but found that the effect of phubbing on relationship satisfaction remained significant independently of relationship duration. Similarly, studies such as Chotpitayasunondh and Douglas (2018), and McDaniel and Coyne (2016) have generally treated relationship duration as a control variable rather than as a focal predictor. As a result, questions such as whether phubbing is perceived as less threatening in long-term relationships or whether perceptions of phubbing decrease as relationship duration increases have not yet been sufficiently examined empirically. A recurring point emphasized across the review of more than 50 studies is that the relationship between relationship duration and the effects of partner phubbing remains underexplored (Roberts and David, 2016; McDaniel and Coyne, 2016). The number of studies directly reporting quantitative analyses of the effect of relationship duration is still limited. On the other hand, meta-analyses and review studies have suggested that the role of relationship duration in the effects of partner phubbing should be examined more systematically.

The findings of the present study are largely consistent with the existing literature, while also offering important points of divergence. In particular, the higher levels of perceived partner phubbing reported by women and married individuals align with previous research (Çizmeci, 2017; Wang et al., 2025). However, the findings related to age and relationship duration provide a more nuanced perspective, especially in light of the mixed results reported in prior studies. This suggests that the impact and perception of partner phubbing may vary depending on relational and contextual dynamics.

From a communication perspective, partner phubbing can be interpreted as a disruption of fundamental interpersonal processes such as attentional engagement, responsiveness, and meaning-making. In face-to-face interactions, individuals rely not only on verbal exchanges but also on nonverbal cues—such as eye contact, body orientation, and attentional presence—to signal involvement and relational value. Phubbing behavior, by redirecting attention toward a mobile device, may function as a nonverbal cue of disengagement, thereby weakening perceived partner responsiveness and emotional connection (Przybylski and Weinstein, 2013; Miller-Ott and Kelly, 2015).

Moreover, within interpersonal communication frameworks, meaning is co-constructed through interaction. Thus, phubbing is not merely a behavior but also a communicative act that is interpreted by the partner. Individuals may attribute meanings such as “I am not important” or “I am being ignored” to their partner’s phone use, which in turn shapes emotional and relational outcomes. This process highlights the role of subjective interpretation and relational meaning-making in understanding the impact of partner phubbing.

In this regard, the present findings—particularly the differences observed across gender, marital status, and relationship duration—can be understood in terms of variations in communication expectations and relational sensitivity. For instance, individuals who place greater importance on responsiveness and emotional attunement may be more likely to perceive phubbing as a relational violation.

The results of the multiple regression analysis showed that perceived partner phubbing was significantly predicted by gender, whereas among relationship duration categories, only the 5–10 years group emerged as a significant predictor. Marital status, age group, and the remaining relationship duration categories were not significant predictors in the regression model. Specifically, male participants reported lower levels of perceived partner phubbing than female participants. This finding may indicate that women are more sensitive to partner phubbing behaviors or may perceive interruptions caused by smartphone use as more disruptive within romantic relationships. In addition, participants who had been in a relationship for 5–10 years reported higher perceived partner phubbing than those who had been in a relationship for 1–12 months. This result suggests that partner phubbing may become more noticeable as relationships progress and daily interaction routines become more established. However, because the other relationship duration categories were not significant predictors, this finding should be interpreted cautiously. Overall, although the regression model was statistically significant, the amount of explained variance was relatively low, indicating that perceived partner phubbing may also be influenced by other relational, psychological, and contextual factors not included in the present study.

## 5 Conclusion

In conclusion, this study revealed that perceived partner phubbing levels differed significantly by gender, marital status, age, and relationship duration. Women, married individuals, participants aged 26 and above, and those in longer-term relationships reported higher levels of perceived partner phubbing. These findings provide important insights into the potential negative effects of technology use and phubbing behavior on relationship quality in romantic relationships.

The findings also highlight the importance of addressing technology use within psychological counseling and couple therapy services. In particular, the higher perception of phubbing among married individuals and those in long-term relationships suggests the risk of what may be described as habit-based invisibility within the relationship. In this regard, the development of a Digital Boundary Agreement for couples may be recommended. Such an agreement may include mutually established rules such as creating phone-free time during meals, putting the screen away while the partner is speaking, or limiting phone use in the bedroom. In addition, awareness of the effects of technology use on romantic relationships may be enhanced by incorporating modules on digital fidelity and digital attention into premarital education programs.

In couple therapy, it is also important to address perceived neglect and micro-emotional disconnections more explicitly. For some individuals, phubbing behavior may be interpreted as a message of worthlessness, and this cognitive framework may be explored in therapy. In this context, partners may be helped to develop communication skills such as active listening, maintaining eye contact, providing empathic feedback, and cultivating mindful attention.

For future research, longitudinal studies are recommended in order to examine changes in phubbing behavior over time. In addition, comparing perceived partner phubbing with actual phone use data such as screen-time records may help reveal the discrepancy between perception and behavior. Furthermore, the development of structured intervention programs aimed at reducing phubbing behavior and the testing of their effectiveness through experimental studies would make important contributions to the literature.

The findings of this study should be interpreted in light of several methodological limitations. First, the reliance on self-report measures may have introduced biases such as social desirability and subjective interpretation, as perceived partner phubbing reflects individuals’ perceptions rather than objective behavior, potentially leading to discrepancies between perceived and actual partner actions. Second, the cross-sectional design limits the ability to draw causal inferences; therefore, future research employing longitudinal designs would provide a more comprehensive understanding of how phubbing behaviors and perceptions evolve over time.

Third, the use of convenience sampling and the overrepresentation of female participants (80.94%) limit the generalizability of the findings. The gender imbalance may have inflated the observed gender effect size, as women tend to be more sensitive to disruptions in partner responsiveness (Miller-Ott and Kelly, 2015; Wang et al., 2025). Additionally, the study relied solely on self-reported perceived partner phubbing without including partners’ actual phone use or dyadic data, restricting conclusions about reciprocal interaction processes. Future research should employ more representative and gender-balanced samples, and utilize dyadic or observational methods to better capture the complexity of phubbing behaviors in romantic relationships.

## Data Availability

The datasets generated and analyzed during the current study are available from the first author upon reasonable request. Requests to access these datasets should be directed to mkarslan@mersin.edu.tr.
